# Dendritic Signaling in Inhibitory Interneurons: Local Tuning via Group I Metabotropic Glutamate Receptors

**DOI:** 10.3389/fphys.2012.00259

**Published:** 2012-07-09

**Authors:** Olivier Camiré, Jean-Claude Lacaille, Lisa Topolnik

**Affiliations:** ^1^Department of Biochemistry, Microbiology and Bioinformatics, Axis of Cellular and Molecular Neuroscience, CRIUSMQ, Université LavalQuébec, PQ, Canada; ^2^Département de Physiologie and Groupe de Recherche sur le Système Nerveux Central, Université de MontréalMontréal, PQ, Canada

**Keywords:** GABAergic interneuron, dendrite, synapse, ion channel, plasticity, metabotropic glutamate receptor

## Abstract

Communication between neurons is achieved by rapid signal transduction via highly specialized structural elements known as synaptic contacts. In addition, numerous extrasynaptic mechanisms provide a flexible platform for the local regulation of synaptic signals. For example, peri- and extra-synaptic signaling through the group I metabotropic glutamate receptors (mGluRs) can be involved in the highly compartmentalized regulation of dendritic ion conductances, the induction of input-specific synaptic plasticity, and the local release of retrograde messengers. Therefore, extrasynaptic mechanisms appear to play a key role in the local tuning of dendritic computations. Here, we review recent findings on the role of group I mGluRs in the dendritic signaling of inhibitory interneurons. We propose that group I mGluRs provide a dual-mode signaling device that integrates different patterns of neural activity. By implementing distinct forms of intrinsic and synaptic regulation, group I mGluRs may be responsible for the local fine-tuning of dendritic function.

## Introduction

Over a century ago, Santiago Ramón y Cajal postulated the law of dynamic polarization, according to which dendrites represent the receiving apparatus of the neuron (Ramón y Cajal, 1891) that integrates the vast majority of synaptic inputs over time. Since then, dendrites have captured the imagination of many researchers. It is difficult not to marvel at the wide diversity and complexity of dendritic arbors, which resemble distinct kinds of trees in a forest. In addition to their morphological complexity, dendrites exhibit a highly complex functional organization. They are endowed with multiple active ion conductances, which control the integration and propagation of local and global dendritic signals in a highly dynamic manner. Two types of signals are generated in dendrites: electrical and chemical. Both can be compartmentalized within individual dendritic branches, providing a means for the synapse-specific integration and modification of incoming information (reviewed in Branco and Häusser, 2010). GABAergic inhibitory interneurons are well known for their heterogeneity at multiple levels, from structural and physiological properties to their corresponding functions in the network. Not surprisingly, interneuron dendrites also exhibit a highly heterogeneous and complex functional organization, which is determined largely by the cell type, the incoming input, and the patterns of ongoing activity. It has been established that an average hippocampal interneuron may receive up to 17,000 synaptic inputs (Gulyás et al., 1999). In addition, a large repertoire of extrasynaptic mechanisms respond to local changes in activity and allow the efficient control of synaptic integration and signal transduction. These mechanisms involve the activation of extrasynaptic glutamate, GABA, acetylcholine, and monoamine and peptide receptors, which can be located in the presynaptic terminals, dendrites, and astrocytes. The mechanisms underlying extrasynaptic signaling in interneurons and its functional role in the modulation of local circuit activity are currently under intensive investigation.

Here, we review recent work that supports the idea that the integrative properties of interneuron dendrites are controlled via activation of group I metabotropic glutamate receptors (mGluRs), which orchestrate a variety of local processes, including calcium signaling, modulation of specific ion conductances, and several forms of synaptic plasticity (Perez et al., 2001; Lapointe et al., 2003; Topolnik et al., 2006, 2009; Galván et al., 2008; Le Duigou and Kullmann, 2011). As local modulation of dendritic function is important for single neuron computations, defining the factors that control dendritic signaling in interneurons will be crucial to understanding interneuron computations. It is not our intention to discuss the important role of group I mGluRs in the regulation of network activity and in different disease states as these topics have been deeply explored in several recent reviews (Nistri et al., 2006; Bartos et al., 2007; Topolnik and Lacaille, 2009; Ribeiro et al., 2010).

## Integrative Properties of Interneuron Dendrites

As in most neurons, the synaptic inputs received by interneuron dendrites are transformed into electrical signals and conducted to the soma. The degree of the signal propagation and its impact on neuronal output are determined by the dendritic architecture and the passive and active properties of dendrites (Geiger et al., 1997; Emri et al., 2001; Nörenberg et al., 2010). In addition, the amplitude and temporal summation of excitatory postsynaptic potentials (EPSPs) are controlled via the activation of local ion conductances, which are often distributed non-uniformly along the somatodendritic axis. For example, direct recordings from dendrites of dentate gyrus basket cells (BCs) and hippocampal *Cornu Ammonis* 1 (CA1) oriens–lacunosum-moleculare (O–LMs) interneurons revealed a relatively constant density of dendritic K^+^ channels (Martina et al., 2000; Hu et al., 2010). The activation of these channels during synaptic depolarization can speed up the time course of EPSPs and, therefore, control the temporal summation of synaptic inputs and the time window for spike generation in interneurons (Fricker and Miles, 2000; Galarreta and Hestrin, 2001). Furthermore, distinct distribution of dendritic Na^+^ channels in BCs vs O–LMs can also affect the amplification of sub-threshold synaptic inputs and spike initiation and propagation. For example, a steep distance-dependent decay of Na^+^ channels is found in dendrites of BCs. Accordingly, Na^+^ spikes can only be initiated in the BC axon but not in its dendrites (Hu et al., 2010). The situation is different however in O–LM interneurons. The estimated density of Na^+^ channels in these cells is threefold larger than in pyramidal cell dendrites (Stuart and Sakmann, 1994; Martina et al., 2000). Accordingly, Na^+^ spikes can be initiated in dendritic sites and can propagate over somatodendritic domain with a relatively constant amplitude and time course.

The propagation of an action potential into neuronal dendrites has a major impact on synaptic input integration and plasticity. These functions of the backpropagating action potential (bAP) are associated with significant depolarization and calcium (Ca^2+^) entry resulting from the activation of voltage-gated Ca^2+^ mechanisms. The properties of bAP-evoked Ca^2+^ transients (bAP-CaTs) vary between types of neurons, depending on dendritic geometry, the properties and the availability of dendritic voltage-gated channels, the endogenous Ca^2+^-binding capacity, and level of activity (Kaiser et al., 2001; Sabatini et al., 2002; Goldberg et al., 2003a; Aponte et al., 2008; Evstratova et al., 2011). However, in most GABAergic interneurons, bAP-CaTs are largely attenuated with distance from the soma (Kaiser et al., 2001; Goldberg et al., 2003a; Evstratova et al., 2011). What can be the factors responsible for such proximal compartmentalization of bAP-CaTs? First, as in most neurons, backpropagation of APs in interneurons is likely to be decremental because of dendritic geometry (Rall, 1964; Goldstein and Rall, 1974; Spruston et al., 1995). The complex dendritic profile of most interneurons, with extensive branching close to the soma, may affect the shape of bAPs and result in rapid branch-dependent bAP attenuation (Rall, 1964; Vetter et al., 2001). Second, the differential subcellular distribution of active conductances and their activity-dependent regulation via extrasynaptic mechanisms play an important role in AP backpropagation (Frick et al., 2004; Sjöström and Häusser, 2006; Hu et al., 2010). Because in most interneurons spike propagation is restricted within proximal dendritic branches, the spike-timing-dependent plasticity regulated by bAPs is likely to occur predominantly at proximal synapses. This raises an important question: what kind of associative signal may operate in distally located synapses of interneurons? One possibility is that local Ca^2+^ spikes can control cooperative plasticity in distal dendrites. Ca^2+^ regenerative events (e.g., Ca^2+^ spikes) have been well characterized in pyramidal neurons, where they require the activation of *N*-methyl-d-aspartate (NMDA) receptors, voltage-gated calcium channels (VGCCs), and Na^+^ channels (Schiller et al., 1997; Larkum et al., 1999; Golding et al., 2002; Losonczy and Magee, 2006). However, the mechanisms underlying Ca^2+^ spike initiation in distal dendrites of interneurons remain unknown.

In summary, despite the lack of the detailed information regarding the subcellular distribution of distinct ion conductances in different types of interneurons, at least two conclusions can be drawn from the findings reported so far. First, the distribution of active ion conductances may vary among different types of interneurons. Second, dependent on the density of voltage-gated Na^+^/Ca^2+^ vs K^+^ conductances, two distinct modes of synaptic integration may operate in interneuron dendrites: compartmentalized (different in proximal vs distal sites or within different dendritic branches) or relatively uniform. Accordingly, distinct types of interneurons will be differentially recruited by the inputs innervating different dendritic regions. Local fine-tuning via extrasynaptic mechanisms may provide additional ways for compartmentalized regulation of synaptic integration. In the following sections we will present the evidence for a compartmentalized regulation of interneuron dendritic conductances and synapse-specific plasticity by group I mGluRs.

## Local Biochemical Signaling via Group I mGluRs

**Figure 1:**
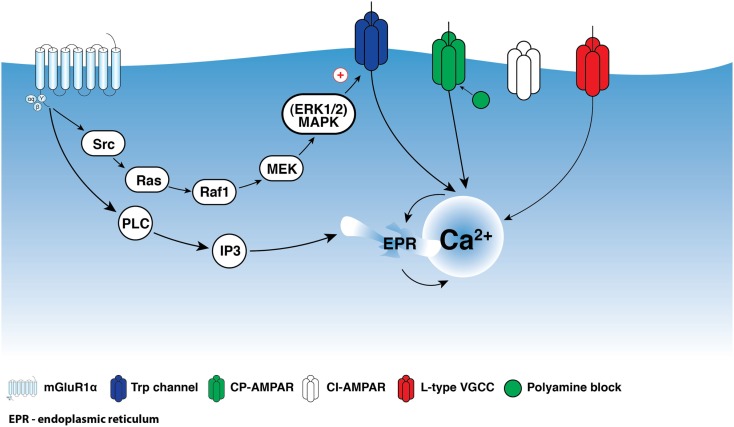
**Schematic representation of mGluR1α signaling in interneuron dendrites**. Activation of mGluR1α is coupled to two parallel signaling pathways: one leading to Ca^2+^ entry via TRP channels and a second producing Ca^2+^ release from intracellular stores. Src/ERK-cascade appears to be involved in the regulation of Ca^2+^ entry via TRP channels, which together with intracellular release are necessary for the induction of Hebbian LTP. In addition, mGluR1α activation together with Ca^2+^ entry through CP-AMPARs appear to be involved in anti-Hebbian LTP induction.

Depending on the level of activity, synaptic inputs can initiate various local biochemical reactions via the activation of synaptic and extrasynaptic signaling mechanisms. In different types of neurons, mGluRs represent the major extrasynaptic signaling platform that couple synaptic activity to various second messengers, including inositol-1,4,5-triphosphate (IP_3_), Ca^2+^, and cyclic adenosine monophosphate (cAMP). Group I mGluRs include two receptor subtypes, mGluR1α and mGluR5, coupled both to the Gα_q/11_ G-protein subunit and to activation of phospholipase C, IP_3_ production, and intracellular Ca^2+^ release (Pin and Duvoisin, 1995; Hermans and Challiss, 2001). In addition, the coupling of group I mGluRs to alternative cascades, such as cAMP-dependent release of arachidonic acid, has been reported (Aramori and Nakanishi, 1992). Group I mGluRs also partake in G-protein-independent signaling via Src-family tyrosine kinases (Heuss et al., 1999; Gee and Lacaille, 2004; Topolnik et al., 2006). For example, in hippocampal CA1 oriens/alveus (O/A) interneurons, activation of mGluR1α triggers membrane depolarization and dendritic Ca^2+^ transients via a G-protein-independent mechanism involving the Src/extracellular signal-regulated kinase (ERK) cascade (Figure 1; Gee and Lacaille, 2004; Topolnik et al., 2006).

Many cell types coexpress mGluR1α and mGluR5, whereas others exhibit specific patterns of distribution of these receptors. In hippocampal CA1 area, mGluR1α is present in several distinct classes of interneurons with their somata located in strata pyramidale, radiatum, and lacunosum moleculare, whereas mGluR5 is expressed uniformly by many cell types (Baude et al., 1993; Lujan et al., 1996; Shigemoto et al., 1997; van Hooft et al., 2000; Ferraguti et al., 2004). However, mGluR1α and mGluR5 may populate distinct dendritic sites (Topolnik et al., 2006). Differential group I mGluR expression was also found in layer IV of the somatosensory cortex, in fast-spiking and regularly spiking interneurons (Sun et al., 2009). Furthermore, using immunogold localization, group I mGluRs have been shown to concentrate in perisynaptic areas (surrounding synaptically located ionotropic receptors) in Purkinje cells (Nusser et al., 1994), hippocampal neurons (Baude et al., 1993; Lujan et al., 1996), neurons of the dorsal horn of the spinal cord (Vidnyánszky et al., 1994), and neurons of the subthalamic nucleus (Kuwajima et al., 2004). The subcellular localization of these receptors is controlled by their direct interaction with Homer proteins (Brakeman et al., 1997).

The recruitment of group I mGluRs depends on both pre- and post-synaptic activity. In hippocampal interneurons, high-frequency repetitive synaptic stimulation is required to evoke responses mediated by group I mGluRs (Topolnik et al., 2005). It has been demonstrated that the inhibition of the activity of astrocytic glutamate transporters, i.e., increase of the availability of extrasynaptic glutamate, facilitates mGluR1α activation in hippocampal O/A interneurons (Huang et al., 2004). Interestingly, mGluR activation in interneurons is also achieved via synaptic stimulation paired with postsynaptic depolarization (Huang et al., 2004; Topolnik et al., 2005). Similar depolarization-dependent activation of mGluR-mediated responses has been shown in pyramidal neurons (Lüthi et al., 1997; Chuang et al., 2000; Rae et al., 2000; Rae and Irving, 2004). The mechanism responsible for the enhancement of mGluR responses through depolarization has yet to be identified in interneurons. The modulation of VGCCs or the activation of non-selective cation currents through the transient-receptor-potential (TRP) channels (Congar et al., 1997; Woodhall et al., 1999; Gee et al., 2003; Topolnik et al., 2006; Hartmann et al., 2008) may be associated with group I mGluR activation. In particular, some members of the canonical subfamily of TRP channels (TRPC1, TRPC4, TRPC5) show a similar voltage-dependence and high Ca^2+^ permeability and are likely to be activated by mGluR1α (Topolnik et al., 2005, 2006).

A hallmark of group I mGluR activation is an increase in intracellular Ca^2+^ concentration resulting from intracellular Ca^2+^ release and/or Ca^2+^ influx through VGCCs or store-operated channels (Pin and Duvoisin, 1995; Hermans and Challiss, 2001). Depending on the cell type and the receptor subtype being activated, group I mGluR-induced intracellular Ca^2+^ elevations may exhibit different temporal properties, varying from plateau-like transient Ca^2+^ rises to Ca^2+^ oscillations. Overall, these Ca^2+^ signals are kinetically slow and may last several seconds. An exemption to this rule is the activation of mGluR1α in O/A interneurons, where it is associated with a relatively fast Ca^2+^ response (Topolnik et al., 2005, 2006). Moreover, mGluR1α/mGluR5-mediated Ca^2+^ signals in these cells can be spatially restricted within individual dendritic branches and play distinct roles in local biochemical signaling (Topolnik et al., 2006). This is in contrast to pyramidal neurons, where group I mGluRs have been involved in the generation of traveling Ca^2+^ waves (Nakamura et al., 1999, 2002; Larkum et al., 2003; Hagenston et al., 2008).

Taken together, these data indicate that biochemical signaling via group I mGluRs is determined by the cell type, the receptor subtype distribution, the local interacting partners, and the conditions of the receptor activation. In interneurons, this signaling can be restricted within individual dendritic branches likely due to a local dendritic geometry and Ca^2+^ buffering. Therefore, in interneurons, group I mGluRs may be well positioned to control the immediate “voisinage” of activated synapses.

## Regulation of Dendritic Conductances via Group I mGluRs

**Figure 2:**
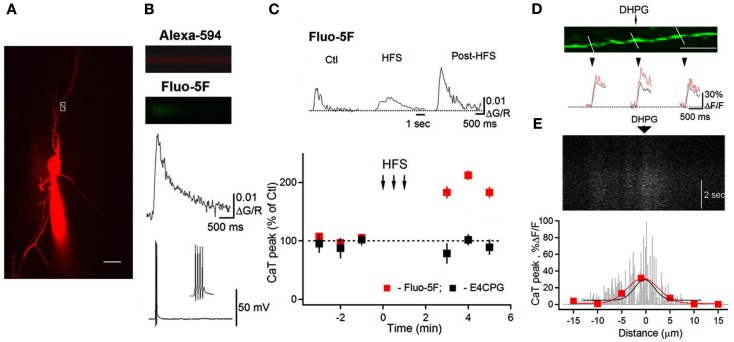
**Potentiation of bAP–CaTs in interneuron dendrites**. **(A)** Two-photon image of an O/A interneuron filled with Alexa 594 and Fluo-5F. White box with a line across the dendrite indicate the position of the line scan to measure bAP–CaT shown in **(B)**. Scale bar, 20 μm. **(B)** Line scan images and associated bAP–CaT. **(C)** Representative bAP–CaTs evoked before (Ctl) during (middle traces; HFS) and after high-frequency synaptic stimulation (post-HFS) within the same dendritic region, and a summary plot, indicating a significant post-HFS potentiation of bAP–CaTs. HFS-induced AP–CaT potentiation was prevented by the mGluR1/mGluR5 antagonist E4CPG. **(D)** Magnified image of a dendrite with lines indicating locations for bAP–CaT measurements. Scale bar, 10 μm. Traces below show bAP–CaTs obtained from these locations in control (black) and after DHPG application (100 μM; red). **(E)** Line scan image collected along the middle part of the dendrite shown in **(D)** and demonstrating a slow DHPG Ca^2+^ response with corresponding spatial profile histogram (black fit). For comparison, the spatial profile of potentiated bAP–CaT (red) obtained from this region is shown superimposed. Modified from Topolnik et al. (2009) with permission.

**Figure 3:**
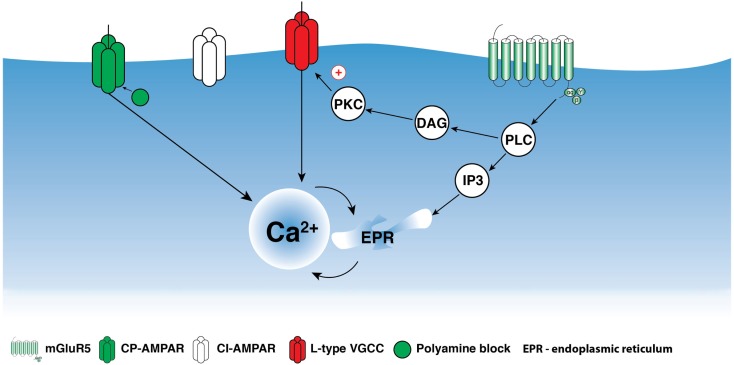
**Schematic representation of mGluR5 signaling in interneuron dendrites**. Activation of mGluR5 may trigger phospholipase C (PLC) activation producing inositol-1,4,5-triphosphate (IP_3_) and Ca^2+^ release from intracellular stores as well as diacylglycerol (DAG)/protein kinase C (PKC) activation. mGluR5-mediated Ca^2+^ release alone can be involved in LTD induction; however, together with Ca^2+^ entry through CP-AMPARs, mGluR5 appears to influence the induction of anti-Hebbian LTP. Moreover, mGluR5-dependent PKC activation produces persistent up-regulation of L-type VGCCs and may play a critical role in the regulation of Hebbian LTP.

The activation of group I mGluRs has a profound effect on the state of ion conductances and on the input–output relationship. The modulation of VGCCs and Na^+^ and K^+^ channels, or channels that mediate non-selective cation currents, has been associated with group I mGluR activation in different cell types including interneurons (Congar et al., 1997; Woodhall et al., 1999; Gee et al., 2003; Huang et al., 2004; Carlier et al., 2006; Ramsey et al., 2006; Topolnik et al., 2006; Hartmann et al., 2008). In O/A interneurons, L-type VGCCs were functionally linked with mGluR5 and played a critical role in regulating the coupling between dendritic APs and Ca^2+^ (Topolnik et al., 2009). bAP-evoked Ca^2+^ entry into dendrites was enhanced for at least 30 min after the activation of mGluR5, but not mGluR1α (Figure 2). This long-lasting increase in bAP Ca^2+^ signaling resulted from the potentiation of L-type VGCCs through Ca^2+^ release from ryanodine-sensitive stores and protein kinase C activation (Figure 3). Importantly, mGluR5 activation and bAP-CaTs potentiation were limited to ~15 μm of dendritic length (Figure 2D), enabling local boosting of bAP Ca^2+^ signaling with a potential effect on the induction of Hebbian long-term potentiation (LTP). A similar form of mGluR – L-type VGCC interaction – exists in cerebellar granule cells (Chavis et al., 1996), which suggests that this mechanism is not limited to O/A interneurons. Such long-lasting potentiation of dendritic Ca^2+^ mechanisms is likely to have an effect on the activation of Ca^2+^-dependent K^+^ channels, with consequences for local input integration and spike generation. Furthermore, several non-specific cation channels or members of the family of TRP channels can be activated by mGluR1α in interneurons (Figure 1; Topolnik et al., 2005, 2006). In addition to their role in synaptic plasticity (see below), these channels provide a persistent membrane depolarization in an input-specific manner, thus shaping local synaptic conductances and controlling the input–output relationship (Egorov et al., 2002).

Together with the highly compartmentalized modulation of dendritic conductances within particular dendritic domains, group I mGluRs may provide global control of information flow via the generation of propagating activity. For example, in pyramidal neurons, mGluR activation is associated with generation of spreading Ca^2+^ waves or regenerative Ca^2+^ events that can control neuronal firing (Larkum et al., 2003; Hagenston et al., 2008). This form of supralinear mGluR signaling is important for overall control of synaptic integration and dendrite-to-soma signaling, but remains to be identified in interneurons.

## Group I mGluRs and Synapse-Specific Plasticity

Because the activation of group I mGluRs is associated with an increase in intracellular Ca^2+^ concentration, the involvement of these receptors in synaptic plasticity has been studied extensively. Several forms of synapse-specific plasticity, including LTP and long-term depression (LTD), that are dependent on group I mGluRs have been discovered at excitatory synapses of cortical interneurons. In a population of O/A interneurons, a form of Hebbian LTP induced by pairing of theta-burst synaptic stimulation (TBS) with postsynaptic depolarization required activation of mGluR1α (Perez et al., 2001; Lapointe et al., 2003; Croce et al., 2010). In addition, this form of LTP was initiated via pharmacological activation of either mGluR1α or mGluR5, indicating the possible independent implication of the two receptors in the induction of this form of plasticity (Le Vasseur et al., 2008). In these cells, mGluR1α activation led to an increase in intracellular Ca^2+^ via the activation of TRP channels and Ca^2+^-induced Ca^2+^ release from ryanodine-sensitive stores (Figure 1), whereas mGluR5 activation was coupled solely to Ca^2+^ release (Figure 3; Woodhall et al., 1999; Topolnik et al., 2006). Interestingly, a similar Hebbian TBS protocol induced LTP in interneurons of the visual cortex, but this LTP was mediated by mGluR5 and not by mGluR1α (Sarihi et al., 2008). Furthermore, although these two examples of LTP required an increase in postsynaptic Ca^2+^, the fact that potentiation was expressed post-synaptically in neocortical interneurons and both pre- and post-synaptically in hippocampal interneurons suggests that these two forms of Hebbian plasticity engage distinct mechanisms.

In addition to the Hebbian form of synaptic plasticity, an anti-Hebbian LTP involving group I mGluRs was reported in a population of hippocampal interneurons (Lamsa et al., 2007; Le Duigou and Kullmann, 2011; Szabo et al., 2012). This LTP was induced by synaptic or pharmacological stimulation at hyperpolarized levels of membrane potential. The anti-Hebbian LTP required the activation of Ca^2+^-permeable α-amino-3-hydroxy-5-methyl-4-isoxazolepropionic acid receptors (CP-AMPARs) and of mGluR1α and mGluR5 (Oren et al., 2009; Le Duigou and Kullmann, 2011). The involvement of group I mGluRs in anti-Hebbian LTP is surprising, as this would require significant excitation of interneurons at hyperpolarized levels of membrane potential. Although such conditions can be met *in vivo*, for example during sharp-wave-associated ripples when O–LM interneurons are silenced but receive a strong excitatory drive from CA1 pyramidal neurons (Klausberger et al., 2003), it is clear that additional mechanisms that are activated at hyperpolarized potentials are likely to cooperate with group I mGluRs during LTP induction. For example, a possible functional interaction may exist between CP-AMPARs and group I mGluRs, as both are located within the same dendritic microdomain (Topolnik et al., 2005). CP-AMPAR Ca^2+^ influx increases slightly with membrane hyperpolarization (Goldberg et al., 2003b; Topolnik et al., 2005), as the CP-AMPAR channel is blocked by endogenous polyamines at depolarizing potentials. Accordingly, mGluR-induced Ca^2+^ release at hyperpolarized levels of membrane potential can lower the threshold for LTP induction (Kullmann and Lamsa, 2007) by increasing the magnitude and duration of dendritic Ca^2+^ elevations. Similar functional interactions exist between mGluRs and TRP channels or VGCCs (Figures 1 and 3; Topolnik et al., 2006, 2009) and are involved in the induction of Hebbian plasticity, suggesting that, regardless of the stimulation paradigm, different Ca^2+^ mechanisms may converge on a common Ca^2+^-dependent signaling cascade, leading to LTP induction.

In addition to LTP, group I mGluRs play a role in the induction of LTD. In hippocampal stratum oriens and radiatum interneurons, application of the group I mGluR agonist (*S*)-DHPG at resting membrane potential resulted in synaptic depression, with mGluR1α inducing reversible depression and mGluR5 inducing long-lasting depression (Le Duigou et al., 2011). In stratum radiatum interneurons, group I mGluR-dependent LTD was also induced by high-frequency synaptic stimulation (Gibson et al., 2008). This type of LTD is likely to be common for synapses formed by Schaffer collaterals, as it was also demonstrated in CA1 pyramidal neurons (Fitzjohn et al., 2001; Huber et al., 2001; Mannaioni et al., 2001). Group I mGluRs also control the direction of plasticity in interneurons. At excitatory synapses of lacunosum-moleculare interneurons in hippocampal region *Cornu Ammonis* 3 (CA3), a protocol that induced Hebbian LTP also induced LTD if mGluR1α was blocked (Galván et al., 2008). The two forms of plasticity required different levels of postsynaptic Ca^2+^ elevation. Whereas L-type VGCCs were involved in both LTP and LTD induction, mGluR1α-dependent Ca^2+^ release in conjunction with L-type VGCC activation was required for LTP. These data indicate that the polarity of plasticity in interneurons can be controlled by specific mGluR1α – L-type VGCC interaction.

Taken together, these findings indicate that the implication of group I mGluRs in the induction of different forms of plasticity in interneurons varies according to the type of interneuron, the stimulation paradigm, the level of membrane potential, the receptor subtype that is activated, and the expression of ionotropic receptors and ion channels that may be part of the mGluR and synaptic compartment. It is worth mentioning that most of the forms of plasticity discussed above were expressed in part presynaptically, implying the group I mGluR-dependent activation of retrograde signaling via yet unknown mechanisms.

## Conclusion

There is growing evidence that group I mGluRs play a critical role in the regulation of dendritic excitability and synaptic plasticity in interneurons. The two receptor subtypes (mGluR1α and mGluR5) have cell type-specific distributions, can be activated by different patterns of synaptic activity, and, via coupling to distinct signaling cascades, can control specific cellular functions (Figures 1 and 3). In particular, mGluR1α can interact with TRP channels, whereas mGluR5 is involved in L-type VGCC modulation, and both receptor subtypes may signal the induction of several forms of synaptic plasticity in interneurons. However, the extent to which the mechanisms activated by the two receptor subtypes are specific to particular types of interneurons, whether these mechanisms reflect the local circuit interactions, and whether they operate under natural conditions *in vivo* remain to be determined.

## Conflict of Interest Statement

The authors declare that the research was conducted in the absence of any commercial or financial relationships that could be construed as a potential conflict of interest.
